# Variation in care and outcome for fragile hip fracture patients: a European multicentre study benchmarking fulfilment of established quality indicators

**DOI:** 10.1007/s00068-024-02549-0

**Published:** 2024-05-31

**Authors:** E Coeckelberghs, K Vanhaecht, A Akiki, P Castillón, B Cox, R El Attal, NB Foss, F Frihagen, TG Gerich, NK Kanakaris, MT Kristensen, M Mohaddes, M Panella, HC Pape, A Sermon, D Seys, S Nijs

**Affiliations:** 1 Leuven Institute for Healthcare Policy, Leuven, KU Belgium; 2 European Pathway Association, Leuven, Belgium; 3 Department of Quality, University Hospitals Leuven, Leuven, Belgium; 4 Hôpital Riviera Chablais, Rennaz, Switzerland; 5 Servei de Cirurgia Ortopèdica i Traumatologia, Hospital Universitari Mútua de Terrassa, Terrassa, Barcelona España; 6 Universitat Autònoma de Barcelona (UAB), Bellaterra, Barcelona España; 7 Klinik für Orthopädie und Unfallchirurgie, Sporttraumatologie, Landeskrankenhaus Feldkirch, Feldkirch, Austria; 8 Departments of Anaesthesia and Intensive Care, Copenhagen University Hospital, Amager-Hvidovre, Hvidovre, Denmark; 9 Denmark Institute for clinical medicine, University of Copenhagen, Copenhagen, Denmark; 10 Orthopedic Department, Østfold Hospital Trust, Grålum, Norway; 11 Institute of Clinical Medicine, University of Oslo, Oslo, Norway; 12 Head of orthopaedic trauma, Centre Hospitalier de Luxembourg, Luxembourg, Luxembourg; 13 Leeds Major Trauma Centre, Leeds Teaching Hospitals NHS Trust, Academic Department of Trauma and Orthopaedics, School of Medicine, University of Leeds, Leeds, UK; 14 Departments of Physiotherapy and Orthopedic Surgery, Copenhagen University Hospital, Amager-Hvidovre, Hvidovre, Denmark; 15 Department of Physical and Occupational Therapy, Copenhagen University Hospital, Bispebjerg-Frederiksberg and Department of Clinical Medicine, University of Copenhagen, Copenhagen, Denmark; 16 Department of Orthopedics, Institute of Clinical Sciences, Sahlgrenska Academy, Gothenburg, Sweden; 17 Department of Orthopedics, Sahlgrenska University Hospital, Mölndal, Sweden; 18 Department of Translational Medicine, University of Eastern Piedmont, Novara, Italy; 19 Department of Trauma, University of Zurich, UniversitätsSpital Zurich, Zurich, Switzerland; 20 Traumatology Department at University Hospitals Leuven, Leuven, Belgium

**Keywords:** Fragile hip fracture, Quality of care, Variation, Care process

## Abstract

**Purpose:**

Despite the availability of clinical guidelines for hip fracture patients, adherence to these guidelines is challenging, potentially resulting in suboptimal patient care. The goal of this study was (1) to evaluate and benchmark the adherence to recently established quality indicators (QIs), and (2) to study clinical outcomes, in fragile hip fracture patients from different European countries.

**Methods:**

This observational, cross-sectional multicenter study was performed in 10 hospitals from 9 European countries including data of 298 consecutive patients.

**Results:**

A large variation both within and between hospitals were seen regarding adherence to the individual QIs. QIs with the lowest overall adherence rates were the administration of systemic steroids (5.4%) and tranexamic acid (20.1%). Indicators with the highest adherence rates (above 95%) were pre-operative (99.3%) and post-operative haemoglobin level assessment (100%). The overall median time to surgery was 22.6 h (range 15.7–42.5 h). The median LOS was 9.0 days (range 5.0–19.0 days). The most common complications were delirium (23.2%) and postsurgical constipation (25.2%).

**Conclusion:**

The present study shows large variation in the care for fragile patients with hip fractures indicating room for improvement. Therefore, hospitals should invest in benchmarking and knowledge-sharing. Large quality improvement initiatives with longitudinal follow up of both process and outcome indicators should be initiated.

## Introduction

Hip fractures have a devastating impact on the elderly and the worldwide incidence will rise, driven by an ageing population (UNFPA, 2011) [1]. Therefore it is important to strive for optimal care which can result in cost-savings and improved patient outcomes [2]. Guidelines and recommendations on the multidisciplinary approach of fragile hip fracture care are available. Moreover, Care Pathways (CPs) are a well-known method to implement guidelines, to improve the quality of care and outcomes by providing a mechanism to better coordinate care and reduce fragmentation [3]. CPs are defined as “complex interventions for the mutual decision-making and organization of care processes for a well-defined group of patients during a well-defined period” [4]. CPs can be a way to facilitate the adherence to guidelines and multidisciplinary collaboration. Contrasting the available evidence to improve care for hip fracture patients, remarkable variation in patient outcomes exists throughout Europe [5]. In addition, several studies have identified practice variance in the treatment procedures of hip fracture patients [6–8]. Despite the availability of clinical guidelines for hip fracture patients, adherence to these guidelines is challenging, resulting in suboptimal patient care [7]. Given the complexity of these frail patients, more integrated care is associated with lower mortality and improvements of quality of care indicators [9]. Moreover, healthcare professionals are often unaware of the actual performance within their organisation. Feedback on the delivered care should provide them guidance in setting hospital-specific improvement initiatives and bridge the gap between guidelines and performed interventions. In that view, a recent study, based on clinical guidelines and expert consensus from 9 European countries provided quality indicators (QIs) for treatment of patients with hip fracture [10]. However, practice testing of these awaits further evaluation.

Therefore, the goal of this study was (1) to evaluate the documentation of and adherence to recently established QIs for rapid recovery of fragile hip fracture patients, and (2) to study clinical outcomes in fragile hip fracture patients from different European countries.

## Methodology

### Study population

This observational, cross-sectional multicenter study was performed in 10 hospitals from 9 European countries (Austria, Belgium, Denmark, Luxembourg, Norway, Spain, Sweden, Switzerland, and the UK). The study was supervised by the European Pathway Association (E-P-A, www.e-p-a.org), an international not-for-profit organization aiming to increase and disseminate knowledge of care pathways. Within the participating hospitals, structural hospital data were collected (Table 1). Further, patient records of consecutive patients were retrospectively collected by a multidisciplinary team. Inclusion criteria were (1) trauma related non-elective admission for hip fracture; (2) minimum age of 65 years; (3) femoral head, femoral neck, trochanteric or subtrochanteric fracture. Exclusion criteria were: (1) hip fracture being not the main reason for admission to emergency room; (2) additional ipsilateral fractures; (3) hip fracture resulting from an in-hospital fall that occurred in the hospital where the patient receives hip fracture care. The local study coordinator was instructed to collect patient record data from the last 30 consecutive patients that were discharged or deceased before May 30th of 2019, using a standardized data extraction form. If data were not available or retrievable in the patient record, this was marked as ‘no information available’. Ethical approval for this study was obtained from the ethical committee of the University Hospital Leuven (S63113). Based on the study protocol, all hospitals provided written agreement of the local study coordinator and approval of the local ethical committee.

**Table 1:** Overview of the (*n* = 21) process indicators, together with the preferred treatment level and the reported timing/duration variable

Indicator	Preferred	Timing/Duration
Pre-operative haemoglobin level assessment	Yes	Time since admission (hours)
Pre-operative cognitive status assessment	Yes	Time since admission (hours)
Pre-fracture mobility status assessment	Yes	Time since admission (hours)
Administration of paracetamol	Yes	Time since admission (hours)
Administration of NSAIDs	No	Time since admission (hours)
Administration of opioids	No	Time since admission (hours)
Administration of nerve blocks	Yes	Time since admission (hours)
Administration of systemic steroids	Yes	Time since admission (hours)
Timing of surgery	In-office hours	Time since admission (hours)
Administration of tranexamic acid	Yes	Time since admission (hours)
Administration of urinary catheter	No	Duration (days)
Administration of wound drain	No	Duration (days)
Intra-operative hypotension	No	Duration (minutes)
Post-operative haemoglobin level assessment	Yes	Time since surgery (hours)
Post-operative pain assessment	Yes	Time since surgery (hours)
Post-operative nutritional assessment	Yes	Time since surgery (days)
Sitting upright in a chair	Yes	Time since surgery (days)
Full weight bearing as tolerated on fracture side	Yes	Time since surgery (days)
Walking > 5 m	Yes	Time since surgery (days)
Indication of start of discharge planning	Yes	Time since admission (days)
Indication of clinical readiness for discharge	Yes	Time since admission (days)

### Variables

Demographic data, QIs regarding the pre-, intra-, and post-operative care and their timings, and data on patient outcomes were collected. A set of QIs that allowed an internationally benchmark of geriatric hip fracture care during hospitalisation was used [10]. That set was developed by combining high levels of evidence with expert opinion. We evaluated 21 process indicators (Table 1) and the following 7 outcomes: delirium during hospitalization, in hospital falls, urinary tract infection, pressure ulcer, surgical site infection, postsurgical constipation, and mortality within 3 months.

### Statistical analyses

Data were recorded using MS Excel®. Analyses were performed using SAS version 9.4. Continuous variables are reported as mean and standard deviation (SD); categorical variables are presented as count and percentage. For each of the (*n* = 21) process indicator we calculated the overall and hospital-specific documentation rates as the percentage of patients for which information on the indicator was available. Adherence to process indicators was defined as the percentage of patients with the preferred treatment for that indicator, with missing information being considered as non-adherence.

## Results

### Patient characteristics

In total, we collected the data of 298 patients fulfilling the in- and exclusion criteria. 9 hospitals delivered data of 30 patients, while 1 hospital delivered data of 28 patients. Patient characteristics are summarized in Table 2. The mean age of the patients was 84.7 years, and 73.2% was female. Fracture type was evenly distributed between intra-and extracapsular fractures. Most patients (67%) were living at home alone or with a caregiver / family member / partner.

**Table 2:** Patient characteristics (*N* = 298)

Characteristic	Mean ± SD or *N* (%)			
Age (in years)	84.7 ± 7.6			
Gender				
Male	80 (26.8)			
Female	218 (73.2)			
BMI	23.7 ± 4.4			
Number of comorbidities present on admission	1.8 ± 1.4			
ASA classification				
I	5 (1.7)			
II	84 (28.2)			
III	155 (52)			
IV	37 (12.4)			
Missing data	17 (5.7)			
Fracture type				
Intracapsular	144 (48.3)			
Extracapsular	154 (51.7)			
In- and out-of-office surgery				
In-office hours weekdays (08:00–18:00 h)	152 (51)			
Out-of-office hours weekdays (18:00–08:00 h)	56 (18.8)			
Weekend	82 (27.5)			
Missing data	8 (2.7)			
Pre-fracture residence				
Home, alone	96 (32.2)			
Home, with help	104 (34.9)			
Nursing home	87 (29.2)			
Rehabilitation centre	1 (0.3)			
Other	2 (0.7)			
Missing data	8 (2.7)			
Pre-fracture mobility status	Bed^a^	In-house^b^	Out-house^c^	Shopping^d^
No difficulty	143 (48.0)	110 (36.9)	70 (23.5)	58 (19.5)
With an aid	41 (13.8)	96 (32.2)	61 (20.5)	31 (10.4)
With help from another	45 (15.1)	25 (8.4)	25 (8.4)	21 (7.1)
Not at all	0 (0.0)	8 (2.7)	42 (14.1)	67 (22.5)
Missing data	69 (23.2)	59 (19.8)	100 (33.6)	121 (40.6)
Pre-fracture Parker, New Mobility Score^e^				
< 5	72 (24.2)			
≥ 5	103 (34.6)			
Missing data	123 (41.3)			

^a^ Able to get in and out of the bed

^b^ Able to get about in the house

^c^ Able to get about out of the house

^d^ Pre-fracture mobility status: Able to go shopping

^e^ Score of 0 to 9 (with a higher score reflecting better mobility), calculated as the sum of scores on the mobility status questions on in-house, out-house, and shopping mobility, using the following scores: 0 for “not at all”, 1 for “with help from another”, 2 for “with an aid”, and 3 for “no difficulty”

*Abbreviations* SD = standard deviation, N = number

### Documentation of and adherence to process indicators

**Fig. 1:**
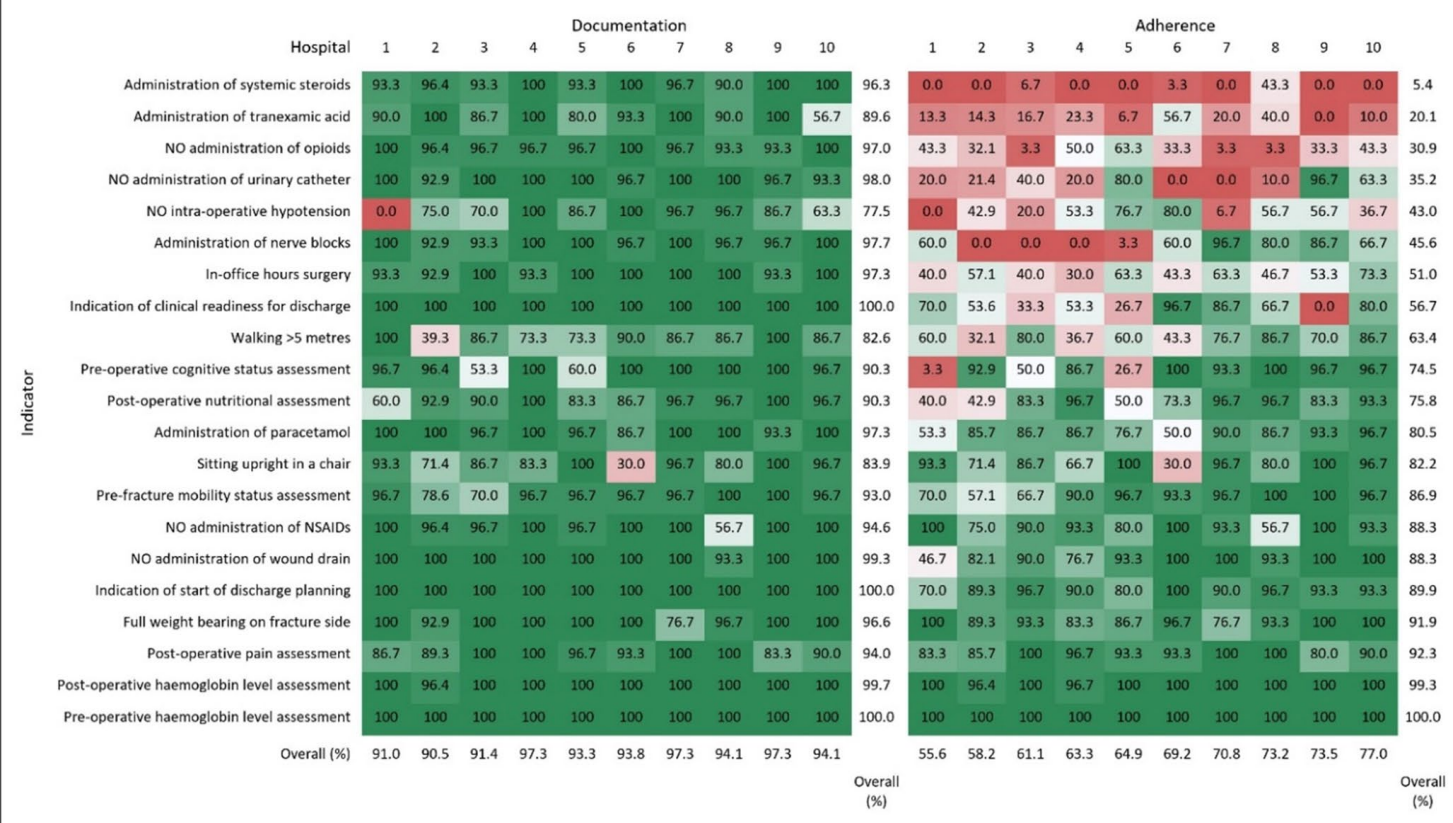
Heat map showing overall and hospital-specific documentation of (left panel) and adherence to (right panel) the 21 process indicators, defined as the percentage of patients with available information and the percentage of patients receiving the preferred treatment, respectively, with red indicating low and green indicating high percentages. Indicators and hospitals are ranked from low to high overall adherence

Figure 1 shows the documentation of and adherence to the 21 process indicators. Complete (100%) documentation was observed for only three indicators: pre-operative haemoglobin level assessment, indication of start of discharge planning, and indication of clinical readiness for discharge. Overall documentation rates were above 90% for all except four indicators: the administration of tranexamic acid (89.6%), intra-operative hypotension (77.5%), walking > 5 m (82.6%), and sitting upright in a chair (83.9%). At hospital-level, overall documentation rates (across indicators) ranged from 90.5 to 97.3%, and indicator-specific rates were mostly above 80%, with no clear outlying hospitals, except for very low rates (< 50%) for three specific hospital-indicator combinations.

Regarding the performance (adherence to the guidelines) of individual QIs, results showed a lot of variation both within and between organisations, also shown in Fig. 1. Overall adherence, i.e. the percentage of patients with the preferred treatment being documented, was below 50% for six indicators (administration of opioids, administration of nerve blocks, administration of systemic steroids, administration of tranexamic acid, administration of urinary catheter, and intra-operative hypotension. Administration of systemic steroids was extremely low, with only 5.4% of patients receiving this intervention, followed by administration of tranexamic acid (TXA) in 20.1% of the patients and opioid avoidance in 30.9% of the patients. Complete adherence (100%) was only observed for one indicator (pre-operative haemoglobin level assessment). Three other indicators showed adherence rates above 90% (post-operative pain assessment, post-operative haemoglobin level assessment, and full weight bearing as tolerated on fracture side). The administration of nerve blocks is highly variable, with three hospitals administrating nerve blocks in at least 80% of their patients, while another three hospitals using no nerve blocks at all, and one hospital only in 1/30 patients.

### Timing of process indicators

Documentation of the timing of the process indicators was poor, with only half of the indicators having a documentation rate above 80%. Documentation rates were even below 50% for the duration of urinary catheterization, time to post-operative nutritional assessment, and time to full weight bearing as tolerated on fracture side.

**Fig. 2:**
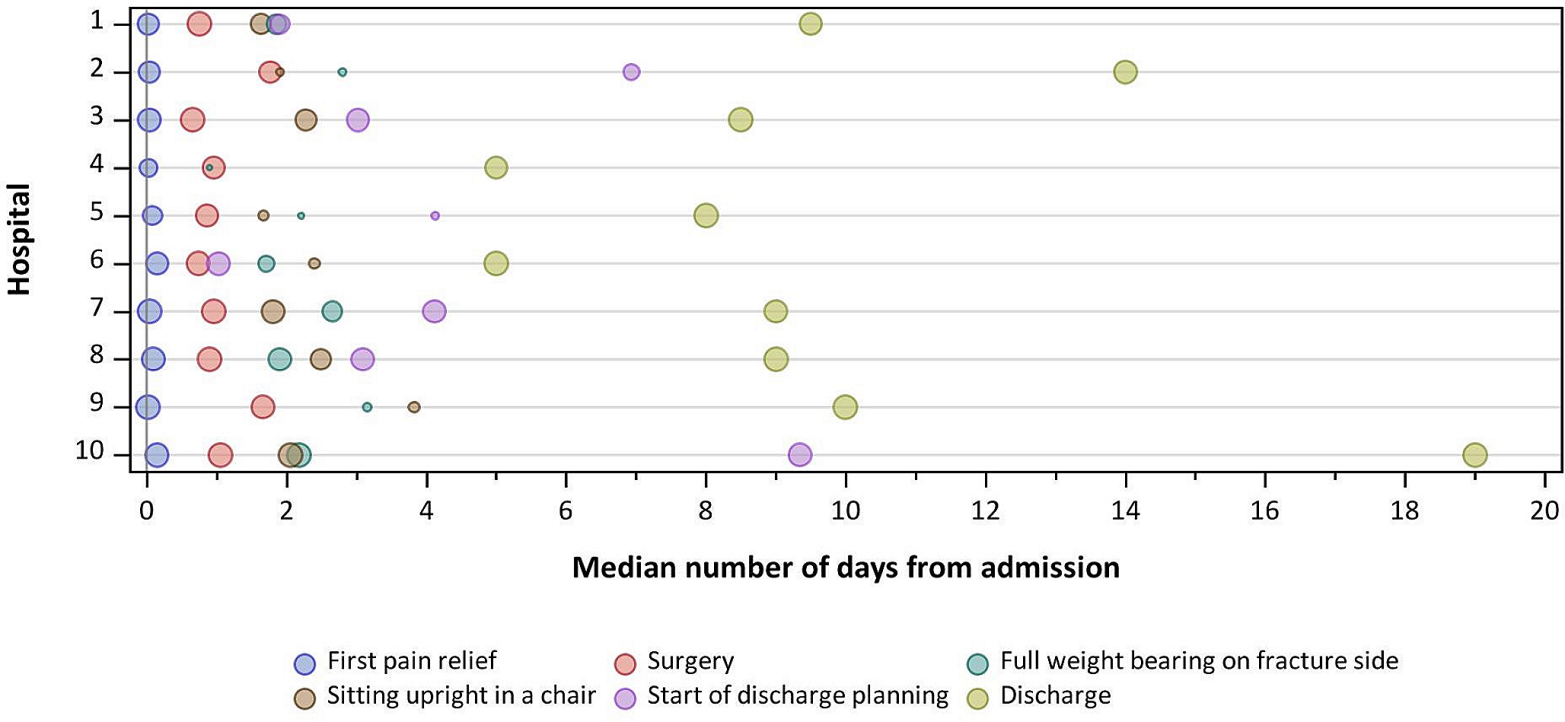
The timing of process indicators relative to the day of admission (timing of admission = 0). The larger the dots, the larger the number of patients for which information on the indicator timing was found in the patient record

Timings and duration of several QIs vary largely between organisations, as shown in Table 3; Fig. 2. Indicators that show large variation between hospitals include assessments of pre-operative cognitive status and pre-fracture mobility status, with median times since admission ranging from 0.1 to 11.8 h and from 0.2 to 18.0 and from hours, respectively. The overall median time to surgery was 22.6 h, with the shortest median time on hospital level being 15.7 h and the longest 42.5 h. The median LOS was 9.0 days and ranged from 5.0 days to 19.0 days.

**Table 3:** Documentation rates of the timing of the process indicators and median timing values, represented as the overall median time across hospitals, together with minimum and maximum hospital-specific median values

Indicator	*N* patients (*N* hospitals)		Documentation (%)	Median time		
	Treatment done^a^	Timing information^b^		Overall	Minimum	Maximum
Time to pre-operative haemoglobin level assessment (hours)	298 (10)	276 (10)	92.6	0.9	0.4	1.9
Time to pre-operative cognitive status assessment (hours)	222 (10)	138 (8)	62.2	2.3	0.1	11.8
Time to pre-fracture mobility status assessment (hours)	259 (10)	135 (8)	52.1	2.2	0.2	18.0
Time to administration of paracetamol (hours)	240 (10)	207 (10)	86.3	2.0	0.5	8.7
Time to administration of NSAIDs (hours)	19 (6)	13 (5)	68.4	3.6	0.4	11.5
Time to administration of opioids (hours)	197 (10)	178 (10)	90.4	2.2	0.7	5.1
Time to administration of nerve blocks (hours)	136 (7)	122 (7)	89.7	4.2	0.4	7.8
Time to administration of systemic steroids (hours)	16 (3)	15 (3)	93.8	20.7	18.5	26.2
Time to surgery (hours)	298 (10)	285 (10)	95.6	22.6	15.7	42.5
Time to administration of tranexamic acid (hours)	60 (9)	54 (9)	90.0	21.7	16.7	56.7
Duration of urinary catheterization (days)	187 (9)	72 (8)	38.5	3.6	1.5	7.0
Duration of wound drain (days)	33 (5)	22 (5)	66.7	1.7	1.1	5.9
Duration of intra-operative hypotension (minutes)	103 (9)	103 (9)	100.0	15.0	10.0	60.0
Time from surgery to post-operative haemoglobin level assessment (hours)	296 (10)	253 (10)	85.5	17.4	12.0	23.1
Time from surgery to post-operative pain assessment (hours)	275 (10)	234 (10)	85.1	3.5	0.4	16.8
Time from surgery to post-operative nutritional assessment (days)	226 (10)	100 (7)	44.2	1.0	0.1	4.0
Time from surgery to sitting upright in a chair (days)	245 (10)	147 (9)	60.0	1.0	0.8	1.7
Time from surgery to full weight bearing as tolerated on fracture side (days)	274 (10)	124 (9)	45.3	1.0	0.6	1.6
Time from surgery to walking > 5 m (days)	189 (10)	96 (8)	50.8	2.1	0.8	2.8
Time to first indication of start of discharge planning (days)	268 (10)	172 (8)	64.2	3.6	1.0	9.3
Time to first indication of clinical readiness for discharge (days)	169 (9)	122 (7)	72.2	8.0	4.1	15.7
Length of stay (days)	298 (10)	286 (10)	96.0	9.0	5.0	19.0

^a^ Number of patients (hospitals) for which the corresponding binary variable indicated that the assessment/administration/treatment was performed

^b^ Number of patients (hospitals) for which the timing of the indicator was documented

### Outcome indicators

Delirium during hospitalization and postsurgical constipation were the most common complications, with a prevalence of 23.2% and 25.2%, respectively. Surgical site infection occurred in only 1% of the patients. The Parker, New Mobility Score upon discharge was documented in 43.7% of the patients, with only 6.4% of them having a score ≥ 5. Overall, 30-day post-admission mortality was 7%, and 4.4% of the patients died between 30 days and 3 months after admission. All outcome indicators are shown in Table 4.

**Table 4:** Outcome indicators, represented as overall number (percentage) together with minimum and maximum values across hospitals

Outcome	Value	Overall *N* (%)	Min *N* (%)	Max *N* (%)
Delirium	Yes	69 (23.2)	1 (3.3)	15 (50.0)
	Missing data	13 (4.4)	0 (0.0)	5 (17.9)
In-hospital falls	Yes	13 (4.4)	0 (0.0)	3 (10.0)
	Missing data	5 (1.7)	0 (0.0)	2 (7.1)
Pressure ulcer	Yes	30 (10.1)	1 (3.3)	8 (26.7)
	Missing data	2 (0.7)	0 (0.0)	1 (3.6)
Surgical site infection	Yes	3 (1.0)	0 (0.0)	1 (3.3)
	Missing data	3 (1.0)	0 (0.0)	2 (6.7)
Post-surgical constipation	Yes	75 (25.2)	0 (0.0)	18 (60.0)
	Missing data	11 (3.7)	0 (0.0)	4 (13.3)
Discharge destination	Home, alone	16 (5.4)	0 (0.0)	7 (23.3)
	Home, with help	54 (18.1)	1 (3.3)	10 (33.3)
	Nursing home	88 (29.5)	2 (6.7)	16 (53.3)
	Rehabilitation centre	97 (32.6)	0 (0.0)	18 (64.3)
	Other	39 (13.1)	0 (0.0)	18 (60.0)
	Missing data	4 (1.3)	0 (0.0)	2 (6.7)
Parker, New Mobility Score^a^ upon discharge	< 5	111 (37.3)	1 (3.3)	29 (96.7)
	≥ 5	19 (6.4)	0 (0.0)	10 (33.3)
	Missing data	168 (56.4)	1 (3.3)	29 (96.7)
Change in mobility status from admission to discharge: Able to get in and out of bed	Improvement	5 (1.7)	0 (0.0)	2 (6.7)
	No change	99 (33.2)	1 (3.6)	18 (60.0)
	Deterioration	119 (39.9)	0 (0.0)	21 (70.0)
	Missing data	75 (25.2)	0 (0.0)	27 (96.4)
Change in mobility status from admission to discharge: Able to get about in the house	Improvement	6 (2.0)	0 (0.0)	1 (3.3)
	No change	61 (20.5)	1 (3.6)	10 (33.3)
	Deterioration	162 (54.4)	0 (0.0)	25 (83.3)
	Missing data	69 (23.2)	0 (0.0)	27 (96.4)
Patient deceased	In hospital	6 (2.0)	0 (0.0)	3 (10.0)
	< 30 days after admission	15 (5.0)	0 (0.0)	7 (23.3)
	30 days − 3 months after admission	13 (4.4)	0 (0.0)	4 (13.3)
	Missing data	17 (5.7)	0 (0.0)	12 (40.0)

^a^ Score of 0 to 9 (with a higher score reflecting better mobility), calculated as the sum of scores on the mobility status questions on in-house, out-house, and shopping mobility, using the following scores: 0 for “not at all”, 1 for “with help from another”, 2 for “with an aid”, and 3 for “no difficulty”

*Abbreviations* N = number, Min = minimum, Max = maximum

## Discussion

Hip fracture management is a complex process with a high risk for complications, especially in frail elderly. However, high adherence to evidence-based guidelines can help to standardize the care process, reduce the risk for complications and improve outcomes. The primary goal of the present study was to benchmark and assess the variation in adherence to QIs for rapid recovery of fragile hip fracture patients amongst 10 European trauma hospitals. Secondary goals were to study process indicators and clinical outcomes in this patient population.

First, the lack of documentation of indicators like intraoperative hypotension and time to walking > 5 m postoperatively, was remarkable with data missing in respectively, 22.5% and 17.4% of the patients. It is of utmost importance for organisations to address this bottleneck first, since a better performance starts with better documentation. Figure 1 shows both the documentation and information on the performance of QIs. Additional, Table 3 shows that documentation of the timing of most QIs is lacking. In half of the QIs (11 out of 22) documentation on the timing is unknown in over 50% of the patients.

The results of our study show large variation between and within the hospitals. Overall, the present study shows an average adherence rate of 67.5%. This is slightly higher than the “Quality of healthcare study”, that shows patients receive on average 55% of the recommended care [11]. In comparison, a previous study showed that patients with hip fracture on average received 38.7% of the recommended care [7]. However, it is difficult to compare different studies since they all measure different key interventions and indicators. Moreover, it has been 10 years since data collection of that publication and in the meantime further research and increased awareness impacted the adherence to evidence-based guidelines. Also, our higher adherence could be the result of our selection of centers of excellence, which all have a special interest in hip fracture care management. The results of the present study show that there is an increased adherence to the guidelines, compared to earlier studies, but there is still room for improvement.

Understanding the variance that was shown on the set of evidence-based measures can help hospitals to gain insight on their own care process and set some hospital-specific improvement priorities. Overall, no hospital excels at all indicators, while also none of them performs poorly on all of them. This means that each hospital has some key interventions or indicators where they perform good at and at the same time has some indicators where there is still room for improvement. Five indicators show an average adherence below 50%: administration of TXA, administration of steroids, avoidance of opioid administration, avoidance of urinary catheter insertion and administration of nerve blocks. This is remarkable since pain management is an essential cornerstone in the perioperative treatment of patients with hip fractures. Our results show that 66% of the patients still receive opioids which is in clear contrast with the NICE guidelines [12]. The administration of paracetamol every 6 h preoperatively is recommended, and opioids should only be administered if paracetamol alone does not provide sufficient preoperative pain relief [12]. Research showed that pre-operative opioid use is associated with a greater risk of readmission, post-operative medical and surgical complications, and potential increase in healthcare utilization [13]. Therefore, the decrease of opioid use should be considered in most organisations. Moreover, the administration of nerve blocks shows a very distinct variation between the participating hospitals. Three hospitals do not use nerve blocks as pain relief, while 3 other hospitals use them in over 80% of their patients. Evidence shows that regional blockade reduces pain on movement within 30 min after block placement. Moreover, a reduced risk for pneumonia, decreased time to first mobilization and cost reduction is shown with nerve blocks in hip fracture patients [14]. Every organization should develop and implement a comprehensive analgesia protocol, incorporating regular paracetamol administration, nerve blockade and the minimisation of opioids, from admission until discharge.

Indicators with the lowest overall adherence rates were the administration of systemic steroids and TXA. On average 5.6% of the patients received systemic steroids, while recent evidence shows that the administration can reduce post-operative pain, the prevalence of delirium and the severity of fatigue after hip fracture surgery in older patients, enabling early mobilisation and recovery [15]. Therefore, it is recommended that the administration of systemic steroids, preferable a high dose upon admission, is performed more routinely in patients admitted with a hip fracture. On average 1 patient in 5 of our sample received TXA, with a lot of variation both between and within the centers. This is in line with what can be found in literature, where there is contrasting evidence regarding the effect of TXA. Meta-analyses state that the administration of TXA, given intraoperatively, will reduce the number of transfusions and total blood loss [16]. However, a randomized controlled trial (RCT) showed that TXA indeed significantly reduced total blood loss in extra-capsular hip fractures, but the TXA group showed a higher post-operative mortality [17]. Also, a recent meta-analysis states there is evidence that TXA reduces the need for blood transfusions after hip fracture surgery [18]. However, the small sample sizes of the included studies and low event rates for adverse effects preclude any definitive conclusions regarding the safety can be established [18]. So, the controverse remains regarding the administration of TXA: it reduces blood loss but not necessarily improves outcome. More research on this topic seems to be essential before the use of TXA can be recommended. Regarding patient outcomes, we found a large variation in LOS, with an overall median of 9.0 days, ranging from 5.0 days to 19.0 days on hospital level. However, it is difficult to compare LOS between hospitals given the international context and different healthcare systems in different countries, with different criteria’s (functional, medically or both) and location for discharging patients. We found that mobility was largely reduced upon discharge compared to pre-fracture and that many patients weren’t discharged directly to their original residence. The loss of pre-fracture mobility status at time of discharge should be given a high attention as this has been associated with increased risk of infection, re-admission, and mortality within the following 30 days [19]. Other research does show that a short time to surgery and avoidance of surgical complications have a positive effect on LOS [20]. In time to surgery, on the other hand, less variation was found. Nine out of 10 hospitals operated their patients on average within 48 h, with 3 hospitals having an average time to surgery of less than 24 h which is in line with the guidelines [21]. Research showed that every hour of delay increased mortality risk, and the association with mortality became statistically significant when delaying surgery over 24 h [21]. This was confirmed in other studies, which showed that early surgery improves outcome and reduces mortality, morbidity, pressure ulcers, infection, and LOS [22]. Which leads us to the mortality rate of our study. In our sample, 21 patients (7%) died within 30 days after admission and another 13 patients (4.4%) between 30 days and 3 months after admission. This is comparable with what can be found in literature, with 30-days mortality rates of 6.8–10% [23, 24]. A meta-analyses evaluating mortality rates of hip fracture patients found that 1-month mortality was 13.3% [25]. However, mortality rates should be interpreted with caution giving the relatively small sample sizes per hospital in this study. Moreover, preoperative condition of the patient should be taken into account when reporting mortality rates.

## Conclusion

The present study shows large variation in the care for patients with fragile hip fractures, with a high adherence for some quality indicators, but there is still room for improvement. Therefore, hospitals should invest in benchmarking and knowledge-sharing. Large quality improvement initiatives with longitudinal follow up of both process and outcome indicators should be initiated. The goal of these improvement collaboratives should be on standardizing care processes and optimize quality of care.

## Data Availability

No datasets were generated or analysed during the current study.
